# The accuracy of MRI diagnosis of thumb ulnar collateral ligament injuries over physical examination in clinical decision-making for surgery

**DOI:** 10.1007/s00402-025-05923-8

**Published:** 2025-05-24

**Authors:** Nicolas Orbenes, Philipp Moog, Klaus Woertler, Jan Neumann, Hans-Guenther Machens, Haydar Kükrek

**Affiliations:** 1 Hand Surgery Section, TUM Universitätsklinikum rechts der Isar, Ismaninger Str. 22, 81675 Munich, Germany; 2 Department of Plastic Surgery and Hand Surgery, TUM Universitätsklinikum rechts der Isar, Ismaningerstr. 22, 81675 Munich, Germany; 3 Muskuloskelettal Radiology Section, TUM Universitätsklinikum rechts der Isar, Ismaningerstr. 22, 81675 Munich, Germany; 4 Department of Radiology, Musculoskeletal Imaging, Cantonal Hospital of Graubünden,, Loëstrasse 170, Chur, 7000 Switzerland

**Keywords:** Ulnar collateral ligament, Stener lesion, Magnetic resonance imaging, Thumb injuries, Diagnostic accuracy, Skier’s thumb

## Abstract

**Purpose:**

This retrospective study assessed whether magnetic resonance imaging (MRI) confers a diagnostic or therapeutic advantage over clinical examination in managing thumb ulnar collateral ligament (UCL) injuries and evaluated its accuracy in lesion characterization.

**Materials and methods:**

We reviewed 96 patients undergoing surgical repair over a ten-year period, 43 of whom had preoperative MRI and 53 who did not.

**Results:**

While MRI exhibited high sensitivity (97%) and specificity (80%) for detecting UCL pathology, its accuracy for differentiating lesion subtypes was only moderate (72–84%). No appreciable difference was noted between MRI and non-MRI cohorts in the proportion of indication-appropriate (57% vs. 45%) or surgeries potentially amenable to conservative treatment (43% vs. 55%).

**Conclusions:**

Thus, MRI did not influence the indication for surgery beyond what was determined by a meticulous physical examination. A thorough clinical assessment remains the mainstay, reserving MRI for diagnostically challenging scenarios or when detailed anatomical visualization is necessary.

**Supplementary Information:**

The online version contains supplementary material available at 10.1007/s00402-025-05923-8.

## Introduction

The ulnar collateral ligament (UCL) is the primary stabilizer of the first metacarpophalangeal (MCP) joint against valgus stress [1]. Injuries to the thumb UCL are common, particularly among athletes, accounting for up to 86% of all injuries to the thumb MCP joint [2]. The incidence of UCL injuries is estimated at 50 per 100,000 individuals annually [3], and are most likely to involve the ulnar collateral ligament, which is up to ten times more common than radial collateral ligament injuries (RCL) [4].

The typical mechanism involves forced radial deviation of the thumb, often due to a fall on an outstretched hand. Injuries may be non-displaced or displaced, and the distinction is critical to appropriate management strategies [5]. In displaced tears, the adductor aponeurosis may interpose between the torn ligament and its insertion, known as a Stener lesion, which requires surgical intervention [6] since the lack of contact between the ligament ends prevents healing, leading to persistent instability regardless of immobilization duration [7] Surgical treatment is also recommended for displacement greater than 3 mm [8, 9]. Many authors advocate surgical management for complete UCL tears, regardless of displacement [10].

**Fig. 1:**
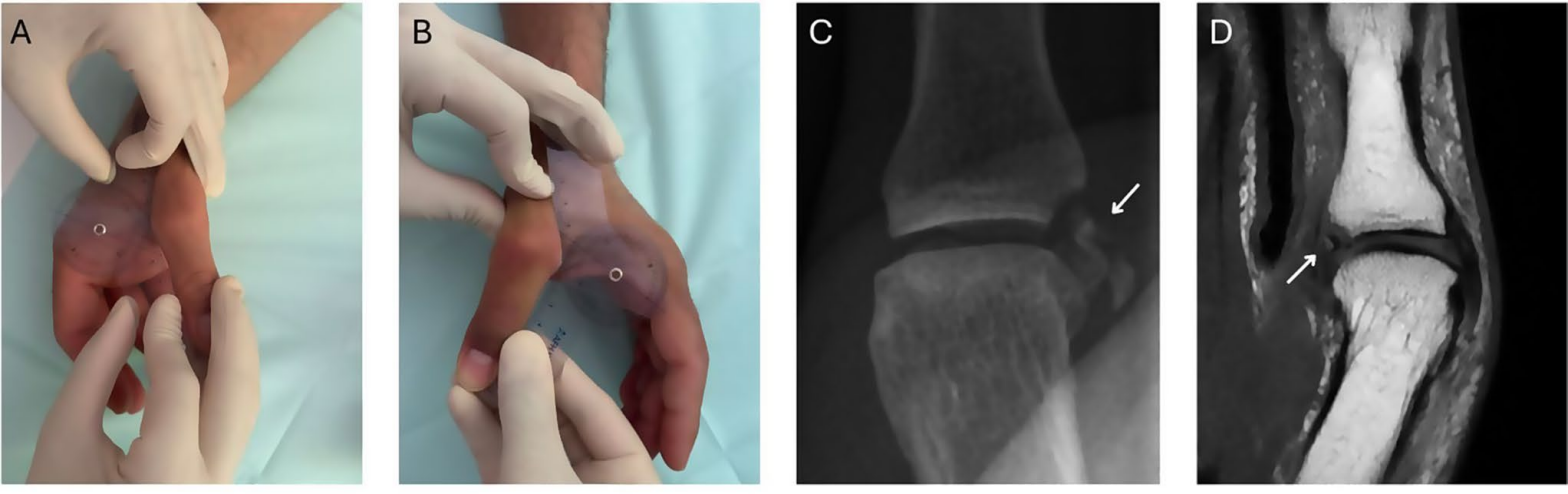
Preoperative photographs of the non-injured and injured side, X-Ray and corresponding MRI picture of a bony UCL-lesion. **(A)** Clinical photograph demonstrating a valgus stress test on the uninjured right thumb MCP joint, showing a physiological joint laxity (valgus opening) of approximately 20°. **(B)** Clinical photograph of the injured left thumb MCP joint under valgus stress, exhibiting pathological instability with a significantly increased valgus opening of approximately 40°, indicative of UCL rupture. **(C)** Preoperative radiograph clearly illustrating a bony avulsion lesion at the UCL insertion site. **(D)** Corresponding MRI scan confirming the presence of the bony avulsion lesion, consistent with radiographic findings and providing detailed visualization of associated soft tissue involvement

Diagnosis of UCL rupture is primarily clinical [11]. However, specificity of physical examination is compromised by pain, swelling, and patient compliance issues during the acute phase [12]. Assessment of thumb laxity is challenging and prone to interobserver variability, with natural side-to-side differences exceeding 15 degrees. Consequently, imaging modalities like X-ray and magnetic resonance imaging (MRI) have become integral in guiding therapeutic decisions (Fig. 1).

MRI has been reported to reliably detect UCL injuries and Stener lesions, demonstrating high sensitivity and specificity [13–16]. However, studies often include small cohorts and lack long-term follow-up, resulting in limited evidence [17]. Furthermore, the added value of MRI over clinical examination remains controversial.

The purpose of this study was to evaluate whether MRI provides advantages over clinical examination in guiding appropriate therapy for thumb UCL injuries and to assess MRI’s accuracy in classifying lesion types compared with intraoperative findings. Physical examination and valgus stress testing remain the cornerstone for diagnosing UCL tears, with > 20° side-to-side difference or > 30° absolute laxity strongly suggesting complete rupture (Fig. 1, Video 1).

## Materials and methods

### Ethics

This study was authorized by the ethics committee of our institution (30.12.2022, 2022-660-S-KK) and was conducted in accordance with the principles of the Declaration of Helsinki. Due to the retrospective nature of the study, the ethics committee waived the need for informed consent.

### Data extraction

We extracted data for patients diagnosed with a UCL tear by filtering for all patients with codes S63.3 - S63.4 according to the international classification of diagnosis coding system (ICD-10) who underwent surgical treatment in the hand surgery department between 2012 and 2022. Information was retrieved from our electronic patients’ records system. The data were exported to plain text and tabulated using Microsoft Excel (Microsoft, Redmond, WA, USA).

### Patient groups

Patients were categorized into two groups based on their preoperative imaging assessment. Group A (*n* = 43) comprised patients who had a preoperative MRI performed either externally or at our institution, while Group B (*n* = 53) consisted of those who proceeded directly to surgery based solely on clinical findings. All patients underwent a standardized clinical examination conducted by a hand surgeon prior to surgical intervention (Video 1). Additionally, routine preoperative radiographs of the thumb MCP joint were obtained for every patient (including those in Group B without MRI) to identify potential bony avulsions and evaluate osteoarthritic changes.

### Surgical indications

The indications for surgery were categorized into absolute and relative indications based on the latest edition of Green’s Operative Hand Surgery [18]. With a trauma history at the thumb and pain at the UCL, a marked instability (> 20° difference to the other side or > 30° absolute laxity) on clinical exam alone was sufficient for surgery decision (Fig. 1, Video 1), even if MRI suggested only partial tearing. When MRI suggested a Stener lesion or a complete tear with gapping of > 2 mm while the clinical exam was not conclusive of a marked instability, the surgery was found to be the appropriate therapy. The aim of the surgery was always to reach a stable MCP-joint on valgus stress in extension as well as 30° flexion (Video 2, Video 3). We did not impose a strict time-based cutoff to distinguish acute from chronic injuries. Instead, in cases with delayed presentation, the absence of relevant osteoarthritic changes on MCP joint radiographs was the key criterion for selecting patients eligible for primary repair. Patients with radiographic evidence of advanced arthrosis were excluded from this cohort, as they were not considered candidates for direct UCL repair. All patients were also counseled preoperatively regarding the potential need for ligament reconstruction (e.g. with a palmaris longus tendon graft) if intraoperative findings revealed irreparable ligament remainders, although none required conversion to reconstruction in this series.

### MRI quality sub-analysis

Two independent expert musculoskeletal radiologists (Analyst 1 and Analyst 2), both blinded to the patients’ clinical information and intraoperative findings, re-evaluated each MRI in a later timepoint postoperatively using predefined image quality criteria in a questionnaire format. These criteria included overall image resolution, appropriate slice orientation relative to the thumb UCL anatomy, adequate field of view to cover the injury region, inclusion of all required pulse sequences, and absence of significant motion artifacts or noise.

### Assessment of surgical appropriateness

**Fig. 2:**
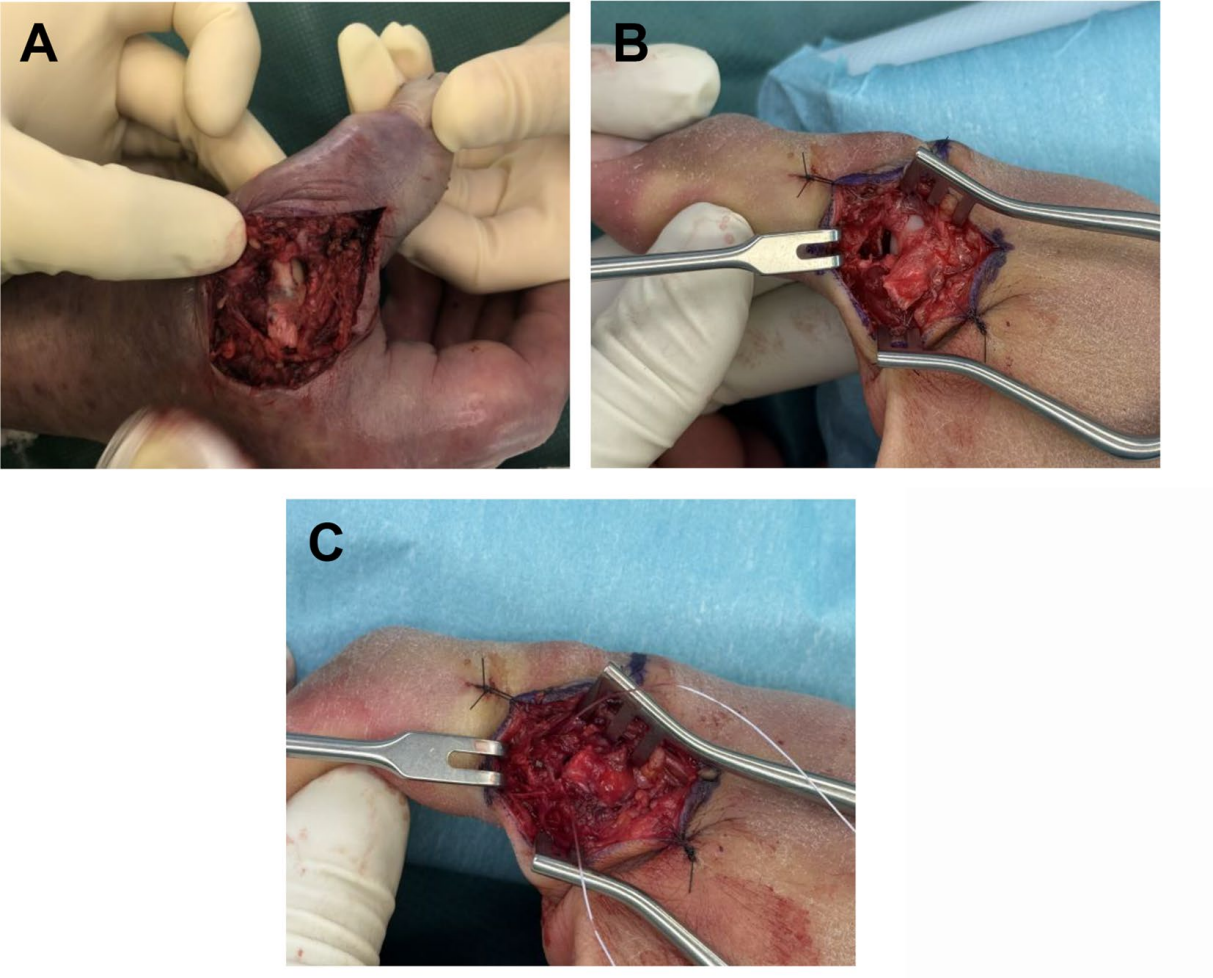
Intraoperative photograph of a complete ligamentous tear of the UCL and a bony avulsion injury of the UCL. **(A)** Intraoperative photograph illustrating a complete rupture of the thumb UCL, with clear retraction of the proximal ligament stump. The exposed articular surface of the first metacarpal bone is visible, providing direct visual confirmation of the high-grade ligamentous lesion previously identified on MRI. **(B)** Intraoperative photograph demonstrating a bony UCL avulsion. The exposed articular surface of the first metacarpal bone and the phalangeal avulsion site is visible (same patient as in Fig. 1). This visual evidence supports the correlation between imaging findings and intraoperative assessment. **(C)** Intraoperative photograph demonstrating the surgical fixation of the bony UCL avulsion, re-establishing anatomical integrity and joint stability

Surgical decisions were classified as “indication-appropriate” if intraoperative findings confirmed the necessity of surgery according to absolute surgery criteria and " potentially amenable to conservative treatment " if the pathology could have been treated conservatively without surgery and a good chance of a good outcome (relative surgery criteria). Stener lesions, bony displaced UCL lesions and complete UCL tears could be seen as absolute criteria (Fig. 2). Partial UCL lesion as well as scar tissue were relative surgery criteria. The impact of adding bony UCL lesions and complete UCL tears to Stener lesions as absolute surgery indications were analyzed separately in both groups and compared to each other. To assess the correctness of surgical treatment, the impact of preoperative MRI (Group A) on the accuracy of the chosen treatment was compared with preoperative clinical evaluation alone (Group B).

### Statistics

The analysis focused on determining whether the use of preoperative MRI influenced the correctness of surgical treatment compared to clinical evaluation alone. Specifically, we compared the number of patients who underwent surgery versus those who underwent surgeries potentially amenable to conservative treatment in both groups (Group A with preoperative MRI versus Group B with clinical assessment only). Fisher’s exact test was used to determine the statistical significance of differences between groups. Odds Ratio (OR) and 95% Confidence Interval (CI) were calculated to assess the association between the use of preoperative MRI and the probability of selecting the appropriate treatment. A p-value of less than 0.05 was considered statistically significant. The radiologists interpreting the MRI were blinded to the clinical exam findings to reduce bias.

## Results

### Demographic characteristics

Our cohort consisted of 197 patients, 96 of whom underwent surgery for suspected thumb UCL rupture. Preoperative MRI scans beside the reports were available for 43 of these patients (Group A), while the remaining 53 patients (Group B) underwent surgery without prior MRI. All patients received preoperative standardized X-ray imaging of the thumb in p.a. and lateral projection. On physical exam all patients had significant instability warranting surgical exploration. In group A, 7 patients received an MRI in-house and 36 patients an external MRI.

The demographic breakdown was 58% male (56/96) and 42% female (40/96), with a mean age of 44 years (SD = 16 years). Injuries were more frequent in the right thumb (58%, 56/96) than in the left thumb (42%, 40/96). The mean time from injury to surgical repair was 48 days, with a range of 1 to 300 days. The most frequent causes were falls, followed by accidents in sports activities, with snow sports injuries among the main causes (Table 1).

**Table 1:** Demographic characteristics and intraoperative findings

	Group A	Group B	Total
**Age at incident (years)**	45 (+/-16)	43 (+/-16)	44 (+/-16)
**Number of Patients**	43	53	96
**Injury to surgery (days)**	50 (1-280)	47 (1-300)	48 (1-300)
**Sex**			
Female	17 (40%)	23 (43%)	40 (42%)
Male	26 (60%)	30 (57%)	56 (58%)
**Hand**			
Left	19 (44%)	21 (40%)	40 (42%)
Right	24 (56%)	32 (60%)	56 (58%)

Age, mean time from injury to surgical repair, gender, side of the ulnar collateral ligament injury of the thumb (shown either as mean +/- standard deviation, range of days or percentage of total)

### MRI and intraoperative findings

Preoperative evaluations using magnetic resonance imaging (MRI) revealed various patterns of ulnar collateral ligament (UCL) injuries of the thumb. Group A (*n* = 43), including all patients with preoperative MRIs, showed the following diagnoses: 5 patients with partial UCL tears, 13 with complete tears, 20 with Stener lesions, 2 with bony skier’s thumb, and 3 with scar tissue without ligamentous discontinuity.

During the surgical exploration of Group A, UCL injuries of the thumb were confirmed in 39 cases, including 8 partial tears, 12 complete tears, 16 Stener lesions, and 3 bony skier’s thumb injuries. In 4 cases, scar tissue or ligament elongation without fiber rupture was found. In cases where MRI suggested a partial tear but clinical instability was pronounced, a complete tear was found, highlighting the priority of physical exam. Lastly, in Group B, which did not rely on MRIs, a total of 48 patients had intraoperative injuries, including 11 partial tears, 17 complete tears, 13 Stener lesions, 7 bony skier’s thumb injuries. The remaining 5 patients had ligament elongation (Table 2).

**Table 2:** MRI and intraoperative findings

		Partial UCL Tear	Complete UCL Tear	Stener Lesion	Bony skier’s thumb	Scar Tissue/ No Injury	Total
Group A	MRI findings	5	13	20	2	3	43
	Intraoperative findings	8	12	16	3	4	43
Group B	Intraoperative findings	11	17	13	7	5	53

MRI and Intraoperative findings of ulnar collateral ligament (UCL) injuries across different groups

### MRI quality assessment by expert radiologists

Due to observed mismatches between the preoperative MRI assessments and intraoperative findings, we performed a focused retrospective quality review of all available external MRI scans. The goal of this sub-analysis was to determine whether suboptimal MRI quality could explain part of the diagnostic discrepancies observed between MRI interpretations and operative findings. Twenty-eight external MRI scans were retrospectively re-evaluated. Analyst 1 judged 13 scans to be of sufficient quality and excluded 15 due to poor resolution, incorrect slice orientation, or missing sequences. Analyst 2 considered 20 MRIs acceptable and excluded 8. The most common reasons for exclusion were low image resolution and inadequate anatomical orientation (Table 3).

**Table 3:** MRI quality assessment

Category	Analyst 1	Analyst 2
Poor Resolution	4	1
Small Field of View (FOV)	1	1
Inadequate Sequence Orientation	7	0
Absence of Necessary Sequences	4	2
Motion Artifacts	1	1
Excessive Noise	0	3
Non-Diagnostic	1	0
**Total Inadequate MRIs**	**15**	**8**
**Total Adequate MRIs**	**13**	**20**

Detailed description of the evaluation by both radiologists

### Diagnostic accuracy of MRI in detecting and differentiating UCL injuries

**Fig. 3:**
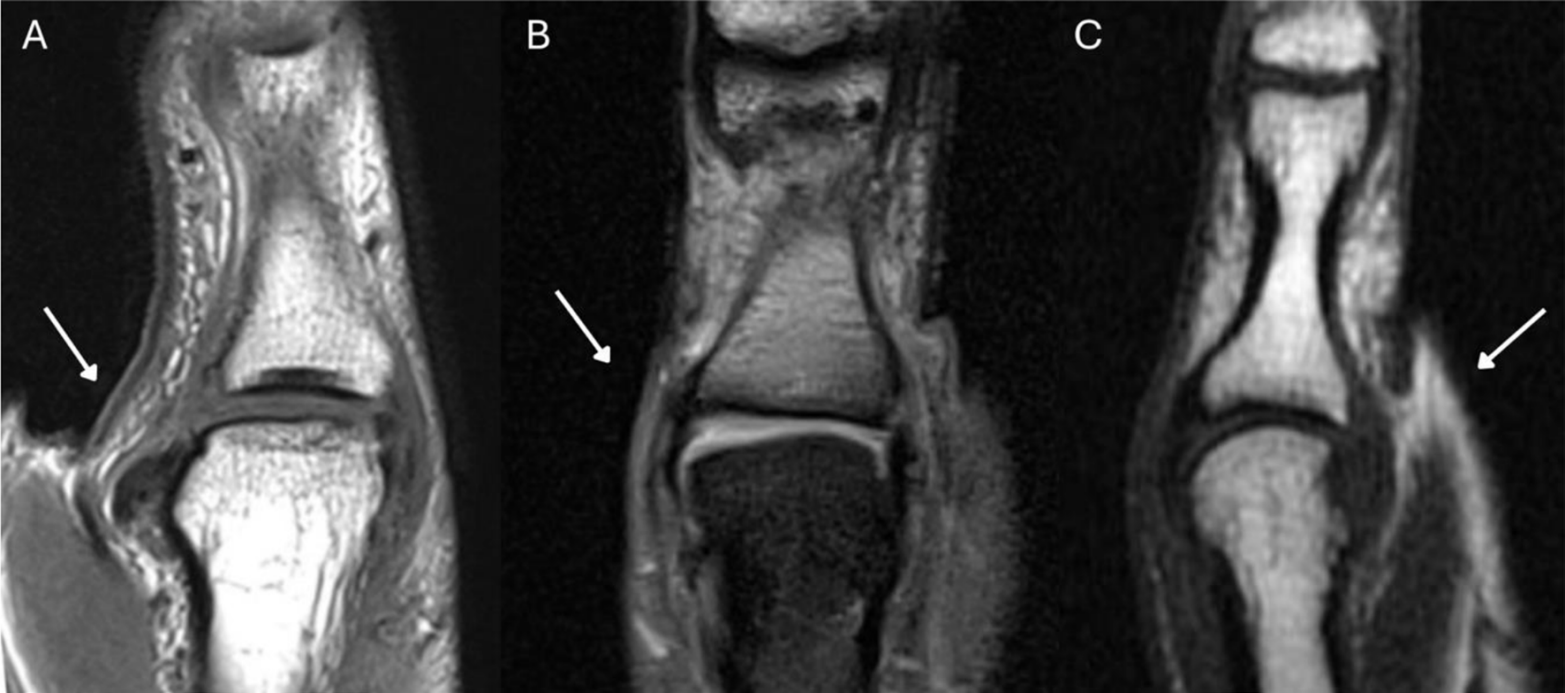
Coronal magnetic resonance imaging (MRI) illustrating three distinct UCL injury patterns. The side-by-side comparison emphasizes the challenges radiologists face when differentiating partial from complete tears. Importantly, although images B and C exhibit distinct radiographic patterns, both lesions were intraoperatively confirmed as complete ruptures of the thumb UCL: **(A)** MRI demonstrating a clear complete rupture of the thumb UCL with an associated Stener lesion, characterized by displacement and interposition of the adductor aponeurosis. **(B)** MRI depicting a complete UCL tear without interposition of the adductor aponeurosis. **(C)** MRI showing an acute UCL injury presenting diffuse edema with suspected partial tear or ligamentous elongation, highlighting the diagnostic ambiguity often encountered during MRI interpretation

MRI demonstrated high diagnostic accuracy in detecting ulnar collateral ligament (UCL) injuries, with an overall sensitivity of 97% and specificity of 80% in the total cohort (*n* = 43). However, its performance in distinguishing specific types of UCL injuries showed variability highlighting the difficulties of MRI interpretation (Fig. 3). For Stener lesions, MRI achieved a sensitivity of 75% and specificity of 70%. Complete UCL tears had a sensitivity of 67% and specificity of 84%, while partial UCL tears were detected with a lower sensitivity (40%) but a high specificity of 97% (Table 4).

**Table 4:** Accuracy of MRI for UCL injury diagnosis

	True Positive (TP)	False Positive (FP)	False Negative (FN)	True Negative (TN)	Sensitivity	Specificity	PPV	NPV	Accuracy
UCL Lesion	37	1	1	4	0.97	0.8	0.97	0.8	0.95
UCL Partial Tear	4	1	6	32	0.4	0.97	0.8	0.84	0.84
UCL Complete Tear	8	5	4	26	0.67	0.84	0.62	0.87	0.79
Stener Lesion	12	8	4	19	0.75	0.7	0.6	0.83	0.72

Efficacy of MRI for diagnosing ulnar collateral ligament injuries of the thumb in group focusing on sensitivity, specificity, positive predictive value (PPV), negative predictive value (NPV) and accuracy

### MRI compared with clinical examination for indication-appropriate therapy

Preoperative MRI was compared to physical examination to evaluate its advantages in guiding indication-appropriate therapy for the surgical management of UCL lesions of the thumb. The goal of the analysis was to determine whether MRI leads to more accurate clinical decision-making, resulting in a higher rate of correctly indicated surgeries, while also assessing if MRI helps reduce surgeries potentially amenable to conservative treatment that could have been managed conservatively.

No significant difference in correct surgical indications was observed between the MRI and non-MRI groups (*p* > 0.05), suggesting that the physical exam alone was sufficient for most decisions (Table 5). Fisher’s exact test showed no statistically significant difference in the correctness of surgical decisions between Group A and Group B (*p* = 0.43). The Odds Ratio (OR) was 1.64 (95% CI: 0.65–4.16), suggesting a potential trend towards more correct decisions in Group A, though this result was not statistically significant. When including displaced bone lesions, Fisher’s exact test again revealed no significant difference in treatment decision correctness between the groups (*p* = 0.80). The Odds Ratio (OR) was 1.27 (95% CI: 0.48–3.36), indicating that preoperative MRI might lead to slightly more correct decisions, but without statistical significance. Finally, when complete UCL tears were included, Fisher’s exact test did not demonstrate a significant difference between the groups (*p* = 0.83). The Odds Ratio (OR) was 1.12 (95% CI: 0.46–2.71), suggesting a slight trend toward improved decision-making with MRI, though again, the result was not statistically significant.

**Table 5:** Surgical decision-making accuracy

	Indication-appropriate Surgery			Avoidable Surgery
	Stener Lesion	Stener Lesion + Bony skier’s thumb	Stener Lesion + Bony skier’s thumb + Complete UCL Tear	Partial UCL Tear or Scar Tissue
Group A	57%	61%	72%	28%
	(16/28)	(19/31)	(31/43)	(12/43)
Group B	45%	56%	70%	30%
	(13/29)	(20/36)	(37/53)	(16/53)

Surgical decision-making accuracy for Stener lesions, with or without bony injuries, with or without UCL complete Tear

## Discussion

Our findings indicate that although MRI is highly sensitive and specific for detecting thumb UCL injuries, it does not significantly improve clinical decision-making over a careful clinical examination. Indication-appropriate surgeries were similar between MRI (57%) and non-MRI (45%) groups (*p* = 0.43), and surgeries potentially amenable to conservative treatment were 43% vs. 55% (*p* = 0.68), respectively. These findings remained non-significant even when focusing solely on Stener lesions or broader absolute indications. Moreover, MRI did not significantly reduce the number of patients potentially treatable without surgery. These results align with previous literature questioning the routine use of MRI [19] and supports evidence indicating that clinical evaluation alone is as effective as MRI for diagnosing skier’s thumb [20]. It is suggested that MRI should be reserved for cases where clinical findings are inconclusive or for complex injuries necessitating detailed anatomical assessment. A meticulous clinical examination remains the cornerstone for the diagnosis of UCL injuries and the determination of surgical intervention [12]. Techniques such as stress testing and the palpation of tenderness or a palpable mass have demonstrated efficacy when conducted by skilled physician’s hands [11, 21]. MRI could reduce uncertainty in complex cases by providing detailed visualization of ligamentous and soft-tissue anatomy. Multiple studies report only marginal benefits of MRI over clinical judgment [22]. Given that clinical examination alone resulted in a substantial proportion of appropriate surgeries, routine MRI may not be necessary, particularly in settings where clinical expertise is high. However, in cases where clinical findings are ambiguous or when surgical planning requires detailed anatomical information, MRI could provide valuable supplementary data. Also MRI can provide important information in case of old instabilities without recent trauma. In case of patients without adequate trauma and instability, the UCL tear could be several years old, in those cases it is important to check for arthrosis of the joint and a direct reconstruction might not be possible. This is important in order to provide adequate treatment and patient information (palmaris longus repair, arthrodesis etc.)

Consistent with previous studies [15, 16, 23], MRI demonstrates high sensitivity (97%) and specificity (80%) for detecting UCL injuries. However, its accuracy in classifying specific lesion types, such as Stener lesions, was moderate in our study (72–84%). This finding aligns with prior research indicating that MRI’s precision in distinguishing lesion subtypes remains limited [8, 24, 25]. Additionally, interobserver variability in interpreting MRI findings, particularly in subtle features such as signal intensity and ligament thickness, further emphasizes these limitations [26]. As a sub-analysis, we retrospectively assessed the quality of the preoperative external MRIs due to noted inconsistencies between imaging and intraoperative findings. Several scans demonstrated technical deficiencies, particularly poor resolution and incorrect anatomical orientation. Nevertheless, diagnostic mismatches also occurred among MRIs rated as adequate by the two expert analysists suggesting that interpretation variability—rather than image quality alone—may also contribute to discrepancies between preoperative imaging and surgical findings, reinforcing the known limitations of MRI in reliably differentiating UCL lesion types (Table 3).

Notably Tresley et al. highlighted MRI’s utility in a multimodality framework (including radiography and ultrasound) for confirming or ruling out Stener lesions, emphasizing that surgical correlation remains paramount in ambiguous cases [27]. Similarly, Mahjan et al. compared the accuracy of clinical examination with MRI in a prospective cohort of 30 patients, ultimately finding that clinical evaluation was sufficient in most instances; MRI was primarily beneficial for equivocal findings or to characterize complex injuries in greater detail [20]. These observations are consistent with our study, underscoring that MRI, while accurate, may not be indispensable when clinical expertise is robust.

Given the limitations of MRI in significantly enhancing the rate of indication-appropriate therapies for UCL injuries of the thumb, a hierarchical diagnostic approach is advisable. Clinical examination remains the primary method for assessing UCL injuries, effectively identifying cases that necessitate surgical intervention or conservative management [28]. In addition, MRI presents logistical and economic challenges, such as high costs, long waiting times and the need for advanced equipment, which can delay treatment and overburden healthcare systems, especially in resource-limited settings [29]. In contrast, ultrasound, though more accessible and less costly, has a high operator dependency, which can limit its applicability in certain contexts [30]. Nonetheless, ultrasound remains a viable alternative in specific cases, provided the operator is adequately qualified [31].

Furthermore, the British Society for Surgery of the Hand (BSSH) recently published guidelines on managing thumb ulnar collateral ligament injuries [32] that align with our findings. These guidelines underscore the importance of a thorough clinical evaluation and timely shared decision-making in the management of thumb UCL injuries. They suggest that patients without significant joint instability often respond well to non-operative measures. In contrast, patients who present with marked laxity may benefit from surgical repair or continued immobilization, with physicians and patients collaboratively choosing an appropriate course of action within the first two weeks following injury. The guidelines also highlight the limited evidence for routine MRI use, recommending that imaging serve as a secondary tool when clinical assessments are inconclusive or when detailed anatomical visualization is needed for surgical planning. Our study echoes these recommendations by advocating a personalized, resource-efficient strategy that relies on expert clinical judgment and keeps the patient at the center of decision-making.

This study presents several limitations. First, being a retrospective analysis, there is an inherent risk of selection bias. Additionally, the relatively small sample size limits the generalizability of the results. The absence of long-term follow-up also restricts our ability to fully assess clinical outcomes and the recurrence rate of treated injuries. The lack of a gold standard for evaluating MRI diagnostic accuracy is another significant limitation, affecting the robustness of our findings. A key limitation is the lack of follow-up for patients who were not operated on, which could have provided valuable insights into the effectiveness of conservative treatments. Prospective, multicenter studies with standardized imaging protocols and validated patient-reported outcome measures would be needed to clarify the long-term clinical implications of different diagnostic strategies.

## Conclusion

While MRI can confirm the diagnosis of complex thumb UCL injuries and provide detailed anatomical information, it does not substantially affect clinical decision-making or decrease surgeries potentially amenable to conservative treatment in most cases. A precise physical examination generally remains sufficient to determine whether surgical intervention is required, making MRI more of a confirmatory tool than a primary diagnostic driver. In light of associated costs, potential delays, and interpretative limitations, we recommend reserving MRI for borderline presentations or complicated injury patterns. Additional prospective studies with long-term outcomes are warranted to refine imaging protocols and inform best practices.

## Electronic supplementary material

Below is the link to the electronic supplementary material.

Supplementary Material 1: Video 1: Clinical examination of a bony UCL-lesion with partial instability: The examination is carried out comparing the non-injured right side to the injured left side. First the stability is tested in 0° extension in the MCP-joint to test the accessory collateral ligaments, which are stable on radial or ulnar force on both sides. Next the proper ulnar collateral ligaments are tested on each side in 30° MCP-flexion with a radial and ulnar force on the MCP joint showing an increased ulnar instability of about 20° on the left thumb compared to the right thumb (same patient as in Fig. 3, Video 2 and Video 3).

Supplementary Material 2: Video 2: Intraoperative clinical examination of an instable bony UCL-lesion before open surgery: Video showing the ulnar instability of the MCP-joint of the left thumb before surgery on ulnar-sided force in 30° flexion (same patient as in Fig. 3, Video 1 and Video 3).

Supplementary Material 3: Video 3: Intraoperative clinical examination of a bony UCL-lesion after open UCL-anchor refixation: Video showing no more increased openability of the MCP-joint of the left thumb after anchor refixation on ulnar-sided force in 30° flexion (same patient as in Fig. 3, Video 1 and Video 2).

## Data Availability

No datasets were generated or analysed during the current study.
